# Breeding sex ratio and population size of loggerhead turtles from Southwestern Florida

**DOI:** 10.1371/journal.pone.0191615

**Published:** 2018-01-25

**Authors:** Jacob A. Lasala, Colin R. Hughes, Jeanette Wyneken

**Affiliations:** Department of Biology, Florida Atlantic University, Boca Raton, Florida, United States of America; Florida State University, UNITED STATES

## Abstract

Species that display temperature-dependent sex determination are at risk as a result of increasing global temperatures. For marine turtles, high incubation temperatures can skew sex ratios towards females. There are concerns that temperature increases may result in highly female-biased offspring sex ratios, which would drive a future sex ratio skew. Studying the sex ratios of adults in the ocean is logistically very difficult because individuals are widely distributed and males are inaccessible because they remain in the ocean. Breeding sex ratios (BSR) are sought as a functional alternative to study adult sex ratios. One way to examine BSR is to determine the number of males that contribute to nests. Our goal was to evaluate the BSR for loggerhead turtles (*Caretta caretta*) nesting along the eastern Gulf of Mexico in Florida, from 2013–2015, encompassing three nesting seasons. We genotyped 64 nesting females (approximately 28% of all turtles nesting at that time) and up to 20 hatchlings from their nests (n = 989) using 7 polymorphic microsatellite markers. We identified multiple paternal contributions in 70% of the nests analyzed and 126 individual males. The breeding sex ratio was approximately 1 female for every 2.5 males. We did not find repeat males in any of our nests. The sex ratio and lack of repeating males was surprising because of female-biased primary sex ratios. We hypothesize that females mate offshore of their nesting beaches as well as en route. We recommend further comparisons of subsequent nesting events and of other beaches as it is imperative to establish baseline breeding sex ratios to understand how growing populations behave before extreme environmental effects are evident.

## Introduction

Increasing global temperatures threaten marine turtle populations [1–4]. Most authors’ concerns grow from consideration of temperature-dependent sex determination (TSD), the mechanism by which incubation temperature of the nest directly impacts the sex of the embryo [5,6]. In marine turtles, warmer incubation temperatures tend to produce females, whereas cooler temperatures tend to produce males [7, 8]. Authors are concerned that higher temperatures will cause such a female bias in sex ratios that populations will face extinction [9,10]. Currently however, the magnitude of the sex ratio skew in adults is unknown due to our limited understanding of the proportion of adult males (and males approaching sexual maturity) [11]. Marine turtle individuals are often widely distributed geographically, outside of the nesting season. Dispersed members of populations make detecting sex ratio issues across populations challenging. In addition, adult males are very difficult to access because they rarely come to land. While a determination of adult sex ratio is beyond reach, a functional alternative, breeding sex ratios (BSR: the proportion of males and females that successfully mate at any time) [12] can be used to identify the minimum number of males and females contributing to populations. By estimating BSR at small, growing nesting aggregations a more thorough proportion of the nesting beach can be assessed than at large nesting beaches, and inferences can be made about the impact of climate change on the population as a whole [13, 14].

The loggerhead sea turtle (*Caretta caretta*) is listed globally as vulnerable by the International Union for the Conservation of Nature (IUCN) [15]. However, along the continental US and adjacent waters in the Northwest Atlantic Ocean, it is listed as threatened [16]. The Northwest Atlantic contains one of only two marine turtle nesting aggregations of greater than 10,000 individuals nesting annually [15, 17]. Florida nesting loggerheads make up approximately 90% of that aggregation [18–20]. Florida Fish and Wildlife Conservation Commission (FWC) estimated that 184,064 loggerhead nests were laid in the 2016 nesting season and the overall nesting trend is an increasing one across the state [21].

Due to their accessibility, nesting females, nest success, and hatchlings are frequently examined and used for demographic studies and population models [22–24]. Data on nesting females and hatchlings are supplemented with in-water capture/recapture and satellite tag studies, which provide additional information on the number of turtles [25,26]. The Turtle Expert Working Group estimated that the female loggerheads return to nest every 2.5 years on average [19]; however, using mark-recapture data over a 20-year data set, Phillips et al. estimated it at an average of 3.2 years for turtles nesting in Southwestern Florida [27]. From tagging and resighting data, it has been estimated that loggerheads lay on average from 3–4.1 nests per season [22, 28, 29] while satellite tagging suggests that within the Gulf of Mexico, the average is closer to 5.4 nests per season [30]. Nest frequency is an important metric because it can be used to calculate how many females nest each year. Unfortunately, information regarding adult male behavior and number is lacking. Many in-water capture studies do not identify the sex of the turtles [31]. Studies that do identify the sex of captured individuals tend to examine juvenile sex ratios [32–35]; or are focused on migration or distribution [36,37]. Consequently, male sea turtles’ reproductive behavior is poorly understood and sex ratio cannot be estimated directly.

A variety of methods have been used to infer aspects of male reproductive behavior. In all seven-extant species of marine turtles, it has been shown that sperm from more than one male can fertilize a single clutch (multiple paternity) [12, 38–43]. Furthermore, in at least one species, a single male may mate with more than one female [44]. Little is known about mate choice, and while direct observations of multiple matings occur [45, 46], assigning which male(s) successfully father young from observed copulations may not be accurate. Hormonal studies suggest that loggerhead males could mate annually [47] and satellite tracking of adult males suggests that about 40% remain close to nesting beaches during a breeding season and may therefore mate more than once [11, 37]. Together, these findings suggest that males contribute to multiple nests during a nesting season and might breed more frequently than females. The number of males fathering each clutch can be determined genetically and used to estimate the minimum BSR [12, 48]. Whether the BSR or reproductive behavior vary among populations is unknown.

The primary goal of this study was to estimate the breeding sex ratio for the loggerhead turtle nesting on a small nesting beach on the southwestern coast of Florida. To this end, paternal genotypes were identified through exclusion analysis and were used to estimate the number of males contributing to this population.

## Materials and methods

**Fig 1:**
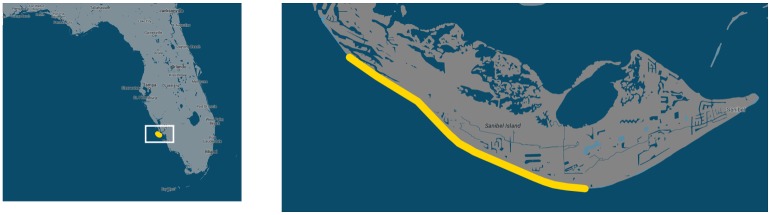
Sanibel Island, Florida. The yellow line notes the 10.3 km (6.4 miles) of beach that is covered in the sampling.

All marine turtle sampling techniques were reviewed and approved by the Florida Fish and Wildlife Conservation Commission (Marine Turtle Permits 13-073A, 14-073D and 15–216) and by the Florida Atlantic University’s Institutional Animal Care and Use Committee (IACUC A13-04). All efforts were made to minimize pain. Nesting loggerhead turtles and their offspring were sampled over three consecutive nesting seasons (late May through July: 2013–2015) on Sanibel Island, in Lee County, Florida. The areas sampled include 10.3 km of beach from J.N. Ding Darling National Wildlife Refuge (26.46719, -82.17030) to Tarpon Bay Road (26.42215, -82.08013) (Fig 1). All nests were monitored by the Sanibel Captiva Conservation Foundation (SCCF) and nesting females were found with their assistance.

Nesting females were identified, measured and tagged during night patrols. To ensure unbiased sample collection from females, nesting turtles were sampled only once. Sampling locations were disinfected with 0.02% chlorohexidine gluconate solution. Blood samples (1 mL per adult turtle) were taken from the external jugular vein [48–50] using a 21G x 1½ inch (3.8 cm) needle and drawn into 4 mL sodium heparin vacutainers (BD Vacutainer Blood Collection Needles/Vacutainers, Franklin Lakes, NJ USA). Skin biopsies, as a backup source of DNA, were taken from the soft tissue along the trailing edge of the pectoral limbs, using either a disposable 6mm biopsy punch (Integra^®^ Miltex^®^, York, Pennsylvania USA) or a sterile one-use razor blade and forceps to collect approximately 6mm^2^ of skin. Skin samples were stored in 70% ethanol until needed for analysis. In rare instances, the female was found returning to the ocean and a blood or skin sample could not be taken. In these cases, one viable egg was collected from her nest immediately. The yolk and albumin were discarded and the eggshell and its eggshell membrane (which are maternally derived) were stored in 70% ethanol (extraction based upon protocols established by [51]).

Nest locations of the sampled females were recorded via GPS and marked following SCCF’s protocols. Screens were placed over research nests to deter predators. Restraining cages were placed over the nest chamber approximately 45 days after oviposition. These cages were used to further prevent predation and ensure an adequate sample of the hatchlings could be obtained upon emergence. Hatchling samples were collected from July through September. Up to twenty hatchlings per nest were collected indiscriminately upon emergence. Blood samples (up to 100 μL per turtle) were taken from the external jugular vein using heparinized syringes (Allergy Syringe with PrecisionGlide^™^ Needle [26G ½ inch] Becton Dickinson, Franklin Lakes, NJ, USA). Skin samples of approximately 1 mm x 3 mm were taken from the trailing edge of one of the flippers using a sterilized scalpel blade. Blood and skin were treated as for nesting females.

All blood samples were stored at -80°C until preparation for DNA extraction. DNA extraction method depended on type of tissue. Blood: 5 μL of whole blood was added to 50 μL of lysis buffer (10mM TRIS, pH8.3, 40mM KCl, 0.5% Tween20 and 200 μg/mL Proteinase K) and incubated at 65°C for 1 h, followed by 100°C for 15 min. Skin/eggshell: a DNEasy blood and tissue kit (Qiagen, Valencia, CA USA) was used following manufacturer′s protocol (for eggshell, incubation was longer than manufacturer’s instructions per [51]). Samples were genotyped with 7 microsatellite loci following published protocols: CcP7E05, CcP2F11, CcP7D04, CcP5H07, CcP7C06, CcP7B07 and CcP8D06 [52]. PCR products were multiplexed together and analyzed with GeneScan500 fluorescent size standard (Applied Biosystems, Foster City, CA USA) using an ABI 3730 DNA Analyzer. Positive and negative controls were run with every PCR plate to identify if there was contamination. Geneious R10 (Biomatters Inc., Newark, NJ, USA) was used to identify alleles. A subset of samples was re-run to identify genotyping error rate. Loci were checked for allelic dropout, stutter and null alleles using MicroChecker 2.2.3 [53].

We assigned paternity through exclusion analysis. Known maternal alleles are “subtracted” from hatchling genotypes and the remaining alleles are assigned to the most likely paternal genotypes. Each female’s alleles and those of her hatchlings were analyzed using COLONY 2.0 [54], a maximum likelihood-based program that determines the maximum number of fathers per clutch based on the genotypes available. Multiple paternity was identified from the presence of three or more paternal alleles identified at each of two or more loci. This approach allows for the possibility of a mutation at one locus [55].

We used GenAlEx [56] to quantify: (1) the observed and expected heterozygosity of alleles and deviations from Hardy-Weinberg Equilibrium (HWE) of the maternal genotypes for each locus, (2) the probability of identity (PI), (3) the probability of exclusion (PE) and (4) F-statistics (F_IS_ and F_ST_). The PI provides the likelihood that two samples will have the exact same genotype given the estimated allelic frequencies at that locus and when all loci are combined in a mixed profile. The PE is the proportion of the population, which has a genotype that contains at least one allele not observed in the mixed profile. The PE value depends on the number of parental genotypes that are known, if only one parent is known (PE_1_), if both parents are known (PE_2_), or if no parents are known (PE_3_). All nests had maternal samples, so we provide PE_1_ for all primers. F_IS_ is equal to the reduction in heterozygosity due to non-random mating within its local population (inbreeding). A positive F_IS_ indicates inbreeding and a negative indicates outbreeding. F_ST_ provides a series of values to identify the genetic structure of a population. Briefly, a value of 0 suggests that the subpopulations are interbreeding freely (panmixis), whereas a value of 1 suggests that the compared subpopulations are genetically distinct.

We modify the calculation of breeding sex ratio [57] using the paternal genotypes (i.e., numbers of males identified = successful males) to estimate the number of breeding males during each year (Eq 1). The equation is used for individual years to compensate for different cohorts of mating and nesting turtles. The typical female loggerhead does not nest annually (usually every 2–3 years), thus, each year of sampling represents a portion of the overall breeding population and the equation can be used for each year separately.

$${Total\;\#\;Breeding\;Males}_{n}\;=\;[(\frac{{total\;\#\;nests}_{n}}{Avg\;\#\;nests\;per\;female\;})x\;({successful\;males}_{n})]$$ (1)

**Eq 1. Equation to determine the minimum number of breeding males**. Where *n* is the year sampled, using the total number (#) of nests, literature values for average number nests/female, and data from this study for the number of males per clutch.

The total number of nests per year is the total number of loggerhead nests laid on Sanibel Island in that year. We include two estimations of the average number of nests per female. The minimum value (3.9) is based off of tagging returns on Keewaydin, a nesting beach south of Sanibel Island [22]. The maximum value (5.4) is based on satellite tag tracking of turtles who returned to nest on beaches north of Sanibel; that value suggests that night patrols are missing re-nesters and underestimate nests/female[30]. Finally, both the estimated number of males and females was rounded to the nearest whole number.

## Results

Over three nesting seasons, 64 females were sampled (2013: 25; 204:16; 2015:23). Thirteen nests were lost due to flooding, predation or did not develop. In those cases, we included the nesting female’s genotype in the allelic frequencies but not in the resulting breeding sex ratios bringing our total to 51 females. In total, 989 hatchlings were sampled: 350 in 2013, 276 in 2014 and 363 in 2015. Of those 51 females, two had been tagged elsewhere (Casey Key and Manasota Key) and four were tagged in Sanibel and returned but we did not resample them.

When the three years were compared, the F_IS_ value was -0.051 (SE: 0.026) and the F_ST_ value was 0.031 (SE: 0.007). These values suggest that there is no evidence of genetic differentiation among yearly cohorts, and therefore they can be considered a single population.

Combining all seven loci resulted in a PI of 7.3 x 10^−13^ and a PE of 1.0. Micro-Checker detected no evidence of scoring error due to stutter, allele dropout, or null alleles. The number of alleles present ranged from 12–24 and there were no deviations from Hardy-Weinberg (p > 0.05). (Table 1).

**Table 1:** Descriptive statistics for each locus.

Locus	N_A_	H_O_	H_E_	PI	PE_1_
P7E	17	0.893	0.913	0.014	0.785
P2F	13	0.889	0.885	0.024	0.726
P7D	14	0.905	0.900	0.018	0.744
P5H	12	0.841	0.858	0.036	0.707
P7C	13	0.952	0.896	0.020	0.726
P7B	18	0.841	0.887	0.021	0.796
P8D	24	0.921	0.943	0.008	0.844

Data from nesting females only. N_A_ is the number of alleles per locus, H_O_ and H_E_ are observed and expected heterozygosities respectively. PI is the probability of identity for each locus, and PE_1_ is the probability of exclusion when one parent is known at each locus.

In 70% of the clutches, more than one male fertilized the clutch (one father per egg so no polyspermy was detected). We identified the genotypes of 126 distinct males. No male fertilized eggs in more than one nest. The maximum number of fathers per nest was 7, mean = 2.51. In 2013, 17 nests were analyzed and 44 fathers were found; in 2014, 14 nests were analyzed and 37 fathers were found and in 2015, 20 nests were analyzed and 45 fathers were found.

Using total numbers of nests (Table 2) and estimates of 3.9–5.4 clutches/female/season [22, 30], we estimate that there were at least 230–318 females nesting on Sanibel from 2013–2015. Based upon these estimates we sampled 20.1–27.8% of the nesting population. Further, our model estimates that there were between 571–829 males mating during that time period. We identified genotypes of 15.2–22.1% of the likely mating males, as male genotypes were unique to each nest analyzed. The BSR for Sanibel Island from 2013–2015 is 1 female: 2.48–2.61 males (Table 2).

**Table 2:** Breakdown of breeding sex ratio (BSR) calculations.

Year	Total # Nests	Estimated # Females	Average # of Males	Estimated # Males
2013	334	62–86	2.58	160–222
2014	411	76–105	3.00	204–321
2015	496	92–127	2.25	207–286
		Σ = 230, 318		Σ = 571, 829
	BSR =	1 Female:		2.48–2.61 Males

Total numbers of nests based upon morning turtle activity censuses, estimated number of females (the minimum values from an average of 5.4 nests per female and the high values from 3.9 nests per female) rounded to the closest individual, average number of males per female (paternity analysis) and the estimated number of males rounded to the closest individual. The number of females and males are summed to reach the BSR value.

**Fig 2:**
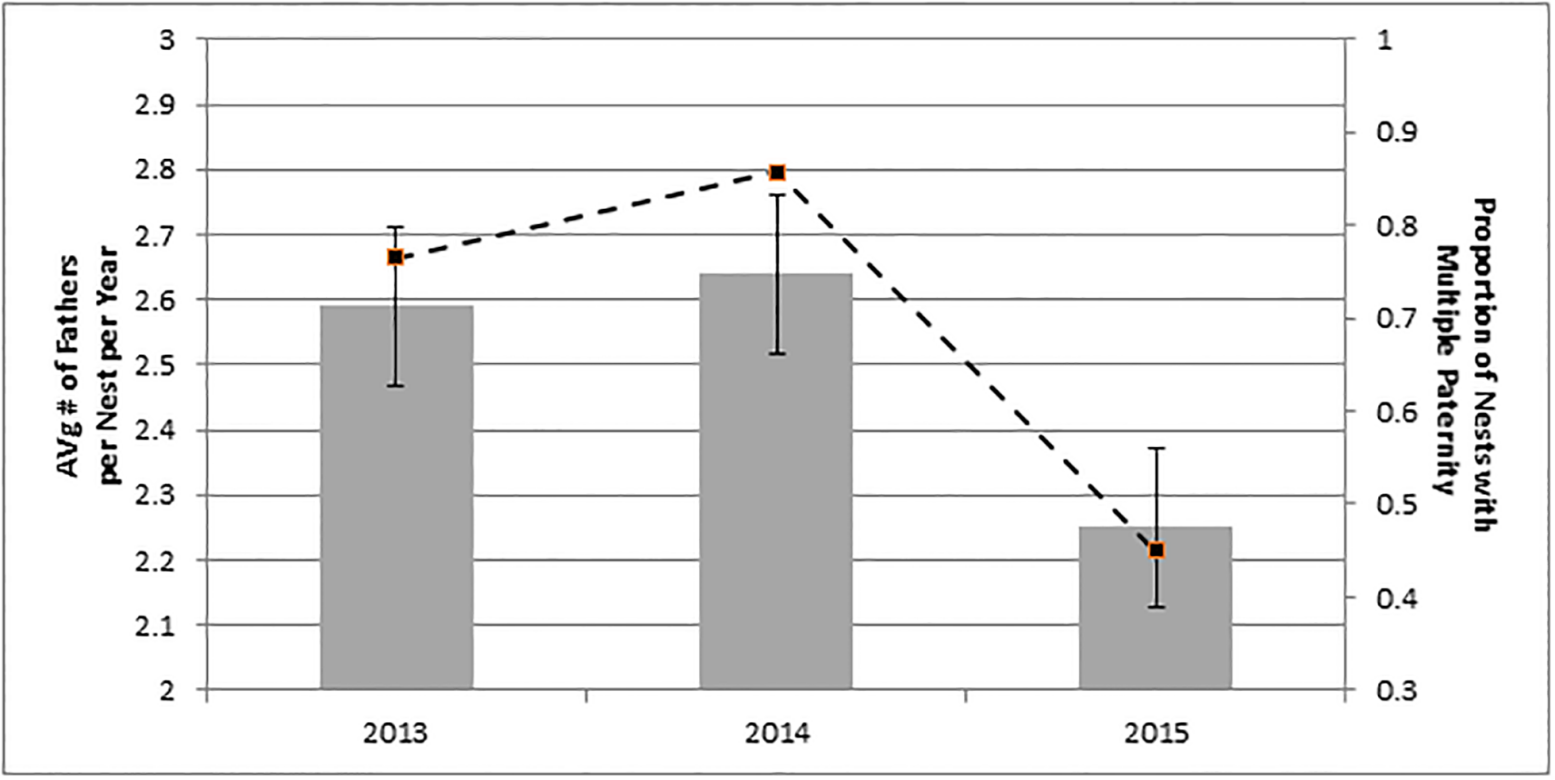
Multiple paternity across seasons. Mean number of fathers/nest/year is depicted by the vertical bars (±standard error of the mean). The proportions of nests with multiple paternity are plotted by the solid dots connected by dashed lines for emphasis (scale on the secondary vertical axis).

In 2015, there were more nests in which a single male fertilized all hatchlings sampled (Fig 2), but we found no statistical difference in number of fathers among years [χ^2^ (df = 2) = 0.905, p = 0.636].

## Discussion

This is the first evidence of multiple paternity in loggerhead sea turtle nests on the Gulf of Mexico coastline. Such behavior is well documented for the loggerheads nesting on the eastern coast of Florida and Georgia [48, 58, 59], as well as other ocean basins [43, 60–62]. To our surprise though, we found that no male had fertilized eggs in more than one clutch within or across years. If males move to breeding areas every year, and some remain for the season [11, 37], it is reasonable to think that a single male would have offspring in more than one female’s nests within a year, even across sequential years. Our result indicates that the number of males contributing to this population of nesting females is higher than would be expected from female-biased primary sex ratios alone [63–65].

The current hypothesis of marine turtle breeding behavior is that females travel to their natal region to mate [66, 67]. Male loggerheads are thought to move close to nesting beaches and compete there to mate with females [68]. Following breeding, males return to foraging grounds and the females remain in the region to nest [69]. In this hypothesis, nesting population structure is driven by natal philopatry of females and gene flow occurs from males that travel along the coast to and from breeding areas [11, 33, 70]. This hypothesis assumes there are female “mating windows” during which males search and compete for females [71] and longer male mating windows. A male that mates earlier therefore, can mate again as soon as opportunity, sperm load, and energy resources allow. If males and female hatchlings and juveniles survive equally, and females return every 2–3 years while males return annually, over a three-year study, at minimum the BSR should approach 1 female for every one male (1:1). Our data do not support this hypothesis because of the numbers of individual males found. By sampling turtles over three years, we were able to estimate the population BSR independent of the complications created by females nesting every second or third year, and males breeding every year.

Here we propose an alternative hypothesis of loggerhead breeding. Female turtles can store sperm after mating events but prior to ovulation; sperm are sequestered in specialized sperm storage tubules [72, 73]. The literature for marine turtles suggests that sperm can be stored for over 3 months [74, 75]. If females mate as they travel from their feeding grounds to their natal regions, they could increase the number of potential mates that they could encounter. In theory, females can be more choosy, without decreasing the likelihood of successful fertilization [76] and the breeding sex ratio will rise as the total number of matings increases. For example, if a female had access to 2 males along the way from her forging area to her nesting grounds, as well as 2 more males in the natal region, the BSR would be 4:1 instead of 2:1. This hypothesis is supported by (i) the large numbers of distinct males found in our study, and (ii) that each males’ genotype occurred just once with and across years. If the pool of available breeding males includes all those that a female encounters along her route to her nesting ground, there is a much-diminished chance of finding one male’s offspring in multiple nests.

Two other kinds of behavior could lead to a larger number of individual males contributing to the population than expected. It is possible that males, like females, do not mate annually [70]; metabolic factors (foraging quality, number of competing individuals, etc.) may make annual breeding impossible. If so, each year’s samples would be a new set of males. Determining whether male behavior changes among years, however, requires formidable efforts beyond the scope of this study (such as coordinated satellite tagging of reproductively active males and subsequent determination of paternity in the offspring of their mates). Alternatively, females may mate throughout their nesting season with a succession of males that arrive at different times. If such were the case, we would not detect that as we did not look at consecutive nests for this study. Future studies will be needed to identify if paternal contributions differ in subsequent nests.

In Florida, the primary sex ratio is already highly female biased, with over 90% female hatchlings produced in some highly productive areas [63, 65, 77], though the effect on adult sex ratio is difficult to predict. As temperatures rise we expect to see more female hatchlings leaving the beach. If those hatchlings survive to adulthood in equal proportions, then the adult sex ratio will skew [23, 78]. It is possible however, that there is differential survival between sexes. One study examined hatchlings that died en route to the ocean from the nest and found that females were four times more likely to perish than their male siblings [79]. Another study examined in-water juvenile sex ratios of loggerheads and found a 2:1 female to male ratio, which was lower than the expected hatchling sex ratio [80]. Further, warmer temperatures produce females, but high temperatures can be fatal to developing embryos and hatching eggs. If temperatures continue to increase, feminized turtles may not survive to leave the nest [4]. Sea turtles are late maturing organisms, so it will take 20 or 30 years of observations to see how breeding sex ratios are affected by current skewed hatchling sex ratios, embryo mortality, and differential hatchling mortality. This study presents results against which those future sex ratios can be compared. If loggerhead hatchling sex ratios have skewed the adult sex ratios, we are not yet seeing the effects in the breeding sex ratio for the Gulf of Mexico. Rather, it seems that females have access to a large number of males, perhaps as they migrate from feeding grounds to the nesting beaches. There is no indication from our data that sex ratio bias has become so severe that extinction risk is elevated.

## References

[1] Santidrian Tomillo P, Saba VS, Lombard CD, Valiulis JM, Robinson NJ, Paladino FV, et al Global analysis of the effect of local climate on the hatchling output of leatherback turtles. Scientific Reports. 2015;5 doi: 10.1038/srep16789 26572897 10.1038/srep16789 PMC4648107

[2] Casale P, Freggi D, Paduano V, Oliverio M. Biases and best approaches for assessing debris ingestion in sea turtles, with a case study in the Mediterranean. Marine Pollution Bulletin. 2016;110(1):238–49. doi: 10.1016/j.marpolbul.2016.06.057 27321803 10.1016/j.marpolbul.2016.06.057

[3] Reneker JL, Kamel SJ. Climate change increases the production of female hatchlings at a northern sea turtle rookery. Ecology. 2016;97(12):3257–64. doi: 10.1002/ecy.1603 27912005 10.1002/ecy.1603

[4] Hays GC, Mazaris AD, Schofield G, Laloe J-O. Population viability at extreme sex-ratio skews produced by temperature-dependent sex determination. Proceedings of the Royal Society B-Biological Sciences. 2017;284(1848). doi: 10.1098/rspb.2016.2576 28179520 10.1098/rspb.2016.2576 PMC5310611

[5] Fuentes MMPB, Fish MR, Maynard JA. Management strategies to mitigate the impacts of climate change on sea turtle′s terrestrial reproductive phase. Mitigation and Adaptation Strategies for Global Change. 2012;17(1):51–63. doi: 10.1007/s11027-011-9308-8

[6] Bachtrog D, Mank JE, Peichel CL, Kirkpatrick M, Otto SP, Ashman T-L, et al Sex Determination: Why So Many Ways of Doing It? Plos Biology. 2014;12(7). doi: 10.1371/journal.pbio.1001899 24983465 10.1371/journal.pbio.1001899 PMC4077654

[7] Bull JJ. Sex determination in reptiles. Quarterly Review of Biology. 1980;55(1):3–21. doi: 10.1086/411613

[8] Mrosovsky N, Yntema CL. Temperature-dependence of sexual-differentiation in sea turtles—implications for conservation practices. Biological Conservation. 1980;18(4):271–80. doi: 10.1016/0006-3207(80)90003-8

[9] Hawkes LA, Broderick AC, Godfrey MH, Godley BJ. Climate change and marine turtles. Endangered Species Research. 2010 p. 137–54.

[10] Jourdan J, Fuentes MMPB. Effectiveness of strategies at reducing sand temperature to mitigate potential impacts from changes in environmental temperature on sea turtle reproductive output. Mitigation and Adaptation Strategies for Global Change. 2015;20(1):121–33. doi: 10.1007/s11027-013-9482-y

[11] Hays GC, Fossette S, Katselidis KA, Schofield G, Gravenor MB. Breeding Periodicity for Male Sea Turtles, Operational Sex Ratios, and Implications in the Face of Climate Change. Conservation Biology. 2010;24(6):1636–43. doi: 10.1111/j.1523-1739.2010.01531.x 20497201 10.1111/j.1523-1739.2010.01531.x

[12] Stewart KR, Dutton PH. Breeding Sex Ratios in Adult Leatherback Turtles (Dermochelys coriacea) May Compensate for Female-Biased Hatchling Sex Ratios. Plos One. 2014;9(2). doi: 10.1371/journal.pone.0088138 24505403 10.1371/journal.pone.0088138 PMC3913748

[13] Dutton DL, Dutton PH, Chaloupka M, Boulon RH. Increase of a Caribbean leatherback turtle Dermochelys coriacea nesting population linked to long-term nest protection. Biological Conservation. 2005;126(2):186–94. doi: 10.1016/j.biocon.2005.05.013

[14] McClenachan L, Jackson JBC, Newman MJH. Conservation implications of historic sea turtle nesting beach loss. Frontiers in Ecology and the Environment. 2006; 4(6):290–6. doi: 10.1890/1540-9295(2006)4[290:ciohst]2.0.co;2

[15] Casale P, Tucker AD. Caretta caretta. The IUCN Red List of Threatened Species. 2015. http://www.iucnredlist.org/details/3897/0.

[16] Federal Register. Endangered and Threatened Species; Determination of Nine Distinct Population Segments of Loggerhead Sea Turtles as Endangered or Threatened. Federal Register; 2011. p. 58868–592.

[17] Turtle Expert Working Group. An Assessment of the Loggerhead Turtle Population in the Western North Atlantic Ocean NOAA Technical Memorandum NMFS-SEFSC-575; 2009. p. 131.

[18] Encalada SE, Bjorndal KA, Bolten AB, Zurita JC, Schroeder B, Possardt E, et al Population structure of loggerhead turtle (*Caretta caretta*) nesting colonies in the Atlantic and Mediterranean as inferred from mitochondrial DNA control region sequences. Marine Biology. 1998;130(4):567–75. doi: 10.1007/s002270050278

[19] Turtle Expert Working Group. An Assessment of the Kemp′s Ridley (*Lepidochelys kempii*) and Loggerhead (*Caretta caretta*) Sea Turtle Populations in the Western North Atlantic. NOAA Technical Memorandum NMFS-SEFSC-409; 1998 p. 96.

[20] Ceriani SA, Meylan AB. Caretta caretta (North West Atlantic subpopulation). The IUCN Red List of Threatened Species. 2015. http://www.iucnredlist.org/details/84131194/0.

[21] Florida Fish and Wildlife Service. 2016 Florida Statewide Nesting Beach Survey Summary. 2016.

[22] Addison DS. Mean annual nest frequency for renesting loggerhead turtles (Caretta caretta) on the Southwest coast of Florida. Marine Turtle Newsletter; 1996. p. 13–5.

[23] Conant TA, Dutton PH, Eguchi T, Epperly, S.P., Fahy CC, et al. Loggerhead sea turtle (Caretta caretta) 2009 status review under the U.S. Endangered Species Act.: Report of the Loggerhead Biological Review Team to the National Marine Fisheries Service.; 2009. p. 222.

[24] Shamblin BM, Dodd MG, Griffin DB, Pate SM, Godfrey MH, Coyne MS, et al Improved female abundance and reproductive parameter estimates through subpopulation-scale genetic capture-recapture of loggerhead turtles. Marine Biology. 2017; 164(6). doi: 10.1007/s00227-017-3166-1

[25] Wibbels T, Owens DW, Morris YA, Amoss MX. Sexing techniques and sex ratios for immature loggerhead sea turtles captured along the Atlantic Coast of the United States. In: Witzell, W.N. (ed.). Ecology of East Florida Sea Turtles. Proceedings of the Cape Canaveral, Florida Sea Turtle Workshop. NOAA Technical Report NMFS-53; 1987. p. 65–74.

[26] Ehrhart LM, Redfoot WE, Bagley DA. Marine turtles of the central region of the Indian River lagoon system, Florida. Florida Scientist; 2007 p. 415–34.

[27] Phillips KF, Mansfield KL, Die DJ, Addison DS. Survival and remigration probabilities for loggerhead turtles (*Caretta caretta*) nesting in the eastern Gulf of Mexico. Marine Biology. 2014;161(4):863–70. doi: 10.1007/s00227-013-2386-2

[28] Murphy TM, Hopkins SR. Aerial and ground surveys of marine turtles nesting beaches in the southeast region, United States. Final Report to U.S. National Marine Fisheries Service, Southeast Fisheries Center, Miami.; 1984. p. 73

[29] LeBuff CR Jr. The loggerhead turtle in the eastern Gulf of Mexico. Sanibel, Florida: Caretta Research Inc p. 216.

[30] Tucker AD. Nest site fidelity and clutch frequency of loggerhead turtles are better elucidated by satellite telemetry than by nocturnal tagging efforts: Implications for stock estimation. Journal of Experimental Marine Biology and Ecology. 2010;383(1):48–55. doi: 10.1016/j.jembe.2009.11.009

[31] Eaton C, McMichael E, Witherington B, Foley A, Hardy R, Meylan A. In-water sea turtle monitoring and research in Florida: review and recommendations. U.S. Department of Commerce, NOAA Technical Memo. NMFS-OPR-38; 2008 p. 233.

[32] Delgado C, Canario AVM, Dellinger T. Sex ratios of loggerhead sea turtles *Caretta caretta* during the juvenile pelagic stage. Marine Biology. 2010;157(5):979–90. doi: 10.1007/s00227-009-1378-8

[33] Arendt MD, Segars AL, Byrd JI, Boynton J, Schwenter JA, Whitaker JD, et al Migration, distribution, and diving behavior of adult male loggerhead sea turtles (*Caretta caretta*) following dispersal from a major breeding aggregation in the Western North Atlantic. Marine Biology. 2012;159(1):113–25. doi: 10.1007/s00227-011-1826-0

[34] Maffucci F, D′Angelo I, Hochscheid S, Ciampa M, De Martino G, Travaglini A, et al Sex ratio of juvenile loggerhead turtles in the Mediterranean Sea: is it really 1:1? Marine Biology. 2013;160(5):1097–107. doi: 10.1007/s00227-012-2160-x

[35] Braun McNeill J, Avens L, Goodman Hall A, Goshe LR, Harms CA, Owens DW. Female-Bias in a Long-Term Study of a Species with Temperature-Dependent Sex Determination: Monitoring Sex Ratios for Climate Change Research. PloS one. 2016;11(8):e0160911–e. doi: 10.1371/journal.pone.0160911 27579608 10.1371/journal.pone.0160911 PMC5007042

[36] Arendt MD, Segars AL, Byrd JI, Boynton J, Whitaker JD, Parker L, et al Distributional patterns of adult male loggerhead sea turtles (*Caretta caretta*) in the vicinity of Cape Canaveral, Florida, USA during and after a major annual breeding aggregation. Marine Biology. 2012;159(1):101–12. doi: 10.1007/s00227-011-1793-5

[37] Arendt MD, Segars AL, Byrd JI, Boynton J, Whitaker JD, Parker L, et al Seasonal distribution patterns of juvenile loggerhead sea turtles (*Caretta caretta*) following capture from a shipping channel in the Northwest Atlantic Ocean. Marine Biology. 2012;159(1):127–39. doi: 10.1007/s00227-011-1829-x

[38] Fitzsimmons NN. Single paternity of clutches and sperm storage in the promiscuous green turtle (Chelonia mydas). Molecular Ecology. 1998;7(5):575–84. doi: 10.1046/j.1365-294x.1998.00355.x 9633101 10.1046/j.1365-294x.1998.00355.x

[39] Kichler K, Holder MT, Davis SK, Marquez R, Owens DW. Detection of multiple paternity in the Kemp′s ridley sea turtle with limited sampling. Molecular Ecology. 1999;8(5):819–30

[40] Jensen MP, Abreu-Grobois FA, Frydenberg J, Loeschcke V. Microsatellites provide insight into contrasting mating patterns in arribada vs. non-arribada olive ridley sea turtle rookeries. Molecular Ecology. 2006;15(9):2567–75. doi: 10.1111/j.1365-294X.2006.02951.x 16842427 10.1111/j.1365-294X.2006.02951.x

[41] Uller T, Olsson M. Multiple paternity in reptiles: patterns and processes. Molecular Ecology. 2008;17(11):2566–80. doi: 10.1111/j.1365-294X.2008.03772.x 18452517 10.1111/j.1365-294X.2008.03772.x

[42] Wright LI, Fuller WJ, Godley BJ, McGowan A, Tregenza T, Broderick AC. No benefits of polyandry to female green turtles. Behavioral Ecology. 2013;24(4):1022–9. doi: 10.1093/beheco/art003

[43] Tedeschi JN, Mitchell NJ, Berry O, Whiting S, Meekan M, Kennington WJ. Reconstructed paternal genotypes reveal variable rates of multiple paternity at three rookeries of loggerhead sea turtles (*Caretta caretta*) in Western Australia. Australian Journal of Zoology. 2014;62(6):454–62. doi: 10.1071/zo14076

[44] Crim JL, Spotila LD, Spotila JR, O′Connor M, Reina R, Williams CJ, et al The leatherback turtle, *Dermochelys coriacea*, exhibits both polyandry and polygyny. Molecular Ecology. 2002;11(10):2097–106. doi: 10.1046/j.1365-294X.2002.01591.x 12296951 10.1046/j.1365-294x.2002.01591.x

[45] Booth J, Peters JA. Behavioral studies on green turtle (Chelonia mydas) in sea. Animal Behaviour. 1972;20(4):808–&. doi: 10.1016/s0003-3472(72)80155-6

[46] Kawazu I, Okabe H, Kobayashi N. Direct observation of mating behavior involving one female and two male loggerhead turtles in the wild. Current Herpetology; 2017 p. 69–72.

[47] Wibbels T, Owens DW, Limpus CJ, Reed PC, Amoss MS. Seasonal-changes in serum gonadal-steroids associated with migration, mating, and nesting in the loggerhead sea turtle (*Caretta caretta*). General and Comparative Endocrinology. 1990;79(1):154–64. doi: 10.1016/0016-6480(90)90099-8 2354777 10.1016/0016-6480(90)90099-8

[48] Lasala JA, Harrison JS, Williams KL, Rostal DC. Strong male-biased operational sex ratio in a breeding population of loggerhead turtles (Caretta caretta) inferred by paternal genotype reconstruction analysis. Ecology and Evolution. 2013; 3(14):4736–47. doi: 10.1002/ece3.761 24363901 10.1002/ece3.761 PMC3867908

[49] Owens DW, Ruiz GJ. New methods of obtaining blood and cerebrospinal-fluid from marine turtles. Herpetologica. 1980;36(1):17–20

[50] Wyneken J. The Anatomy of Sea Turtles. U.S. Department of Commerce NOAA Technical Memorandum 2001 p. 172.

[51] Shamblin BM, Dodd MG, Williams KL, Frick MG, Bell R, Nairn CJ. Loggerhead turtle eggshells as a source of maternal nuclear genomic DNA for population genetic studies. Molecular Ecology Resources. 2011;11(1):110–5. doi: 10.1111/j.1755-0998.2010.02910.x 21429107 10.1111/j.1755-0998.2010.02910.x

[52] Shamblin BM, Faircloth BC, Dodd MG, Bagley DA, Ehrhart LM, Dutton PH, et al Tetranucleotide markers from the loggerhead sea turtle (*Caretta caretta*) and their cross-amplification in other marine turtle species. Conservation Genetics. 2009;10(3):577–80. doi: 10.1007/s10592-008-9573-6

[53] Van Oosterhout C, Hutchinson WF, Wills DPM, Shipley P. MICRO-CHECKER: software for identifying and correcting genotyping errors in microsatellite data. Molecular Ecology Notes. 2004;4(3):535–8. doi: 10.1111/j.1471-8286.2004.00684.x

[54] Jones OR, Wang J. COLONY: a program for parentage and sibship inference from multilocus genotype data. Molecular Ecology Resources. 2010;10(3):551–5. doi: 10.1111/j.1755-0998.2009.02787.x 21565056 10.1111/j.1755-0998.2009.02787.x

[55] Yue GH, Chang A. Molecular evidence for high frequency of multiple paternity in a freshwater shrimp species *Caridina ensifera*. Plos One. 2010;5(9). doi: 10.1371/journal.pone.0012721 20856862 10.1371/journal.pone.0012721 PMC2939052

[56] Peakall R, Smouse PE. GenAlEx 6.5: genetic analysis in Excel. Population genetic software for teaching and research-an update. Bioinformatics. 2012;28(19):2537–9. doi: 10.1093/bioinformatics/bts460 22820204 10.1093/bioinformatics/bts460 PMC3463245

[57] Refsnider JM. High Frequency of Multiple Paternity in Blanding′s Turtle (Emys blandingii). Journal of Herpetology. 2009;43(1):74–81

[58] Bollmer JL, Irwin ME, Rieder JP, Parker PG. Multiple paternity in loggerhead turtle clutches. Copeia. 1999;(2):475–8. doi: 10.2307/1447494

[59] Moore MK, Ball RM. Multiple paternity in loggerhead turtle (Caretta caretta) nests on Melbourne Beach, Florida: a microsatellite analysis. Molecular Ecology. 2002;11(2):281–8. doi: 10.1046/j.1365-294X.2002.01426.x 11856428 10.1046/j.1365-294x.2002.01426.x

[60] Harry JL, Briscoe DA. Multiple paternity in the loggerhead turtle (*Caretta caretta*). Journal of Heredity. 1988;79(2):96–9 3403964 10.1093/oxfordjournals.jhered.a110480

[61] Zbinden JA, Largiader AR, Leippert F, Margaritoulis D, Arlettaz R. High frequency of multiple paternity in the largest rookery of Mediterranean loggerhead sea turtles. Molecular Ecology. 2007;16(17):3703–11. doi: 10.1111/j.1365-294X.2007.03426.x 17845442 10.1111/j.1365-294X.2007.03426.x

[62] Sakaoka K, Yoshii M, Okamoto H, Sakai F, Nagasawa K. Sperm Utilization Patterns and Reproductive Success in Captive Loggerhead Turtles (*Caretta caretta*). Chelonian Conservation and Biology. 2011;10(1):62–72

[63] Wibbels T. Critical approaches to sex determination in sea turtles In: Lutz Musick Wyneken(ed.). The Biology of Sea Turtles, vol 2 Boca Raton, Florida: Taylor and Frances, Ltd/ CRC Press; 2003 p. 103–134.

[64] Rogers M. Hatchling sex ratios and nest temperature sex ratio response of three South Florida marine turtle species (Caretta caretta, Chelonia mydas and Dermochelys coriacea). Boca Raton, Florida: Florida Atlantic University; 2013.

[65] Wyneken J, Lolavar A. Loggerhead sea turtle environmental sex determination: Implications of moisture and temperature for climate change based predictions for species survival. Journal of Experimental Zoology Part B-Molecular and Developmental Evolution. 2015;324(5):465-. doi: 10.1002/jez.b.22635 25877336 10.1002/jez.b.22620

[66] FitzSimmons NN, Limpus CJ, Norman JA, Goldizen AR, Miller JD, Moritz C. Philopatry of male marine turtles inferred from mitochondrial DNA markers. Proceedings of the National Academy of Sciences of the United States of America. 1997;94(16):8912–7. doi: 10.1073/pnas.94.16.8912 9238077 10.1073/pnas.94.16.8912 PMC23194

[67] Bowen B, Avise JC, Richardson JI, Meylan AB, Margaritoulis D, Hopkins-Murphy SR. Population-structure of loggerhead turtles (*Caretta caretta*) in the northwestern atlantic-ocean and Mediterranean Sea. Conservation Biology. 1993;7(4):834–44. doi: 10.1046/j.1523-1739.1993.740834.x

[68] Bowen BW, Karl SA. Population genetics and phylogeography of sea turtles. Molecular Ecology. 2007;16(23):4886–907. doi: 10.1111/j.1365-294X.2007.03542.x 17944856 10.1111/j.1365-294X.2007.03542.x

[69] Bowen BW, Bass AL, Chow SM, Bostrom M, Bjorndal KA, Bolten AB, et al Natal homing in juvenile loggerhead turtles (*Caretta caretta*). Molecular Ecology. 2004;13(12):3797–808. doi: 10.1111/j.1365-294X.2004.02356.x 15548292 10.1111/j.1365-294X.2004.02356.x

[70] Wright LI, Fuller WJ, Godley BJ, McGowan A, Tregenza T, Broderick AC. Reconstruction of paternal genotypes over multiple breeding seasons reveals male green turtles do not breed annually. Molecular Ecology. 2012;21(14):3625–35. doi: 10.1111/j.1365-294X.2012.05616.x 22591073 10.1111/j.1365-294X.2012.05616.x

[71] Fromhage L, Jennions M, Kokko H. The evolution of sex roles in mate searching. Evolution. 2016;70(3):617–24. doi: 10.1111/evo.12874 26842774 10.1111/evo.12874

[72] Gist DH, Congdon JD. Oviductal sperm storage as a reproductive tactic of turtles. Journal of Experimental Zoology. 1998;282(4–5):526–34. doi: 10.1002/(sici)1097-010x(199811/12)282:4/5<526::aid-jez8>3.3.co;2-q 9803538

[73] Sever DM, Hamlett WC. Female sperm storage in reptiles. Journal of Experimental Zoology. 2002;292(2):187–99. doi: 10.1002/jez.1154 11754034 10.1002/jez.1154

[74] Birkhead TR, Moller AP. Sexual selection and the temporal separation of reproductive events—sperm storage data from reptiles, birds and mammals. Biological Journal of the Linnean Society. 1993;50(4):295–311. doi: 10.1111/j.1095-8312.1993.tb00933.x

[75] Sakaoka K, Sakai F, Yoshii M, Okamoto H, Nagasawa K. Estimation of sperm storage duration in captive loggerhead turtles (*Caretta caretta*). Journal of Experimental Marine Biology and Ecology. 2013;439:136–42. doi: 10.1016/j.jembe.2012.11.001

[76] Kokko H, Mappes J. Multiple mating by females is a natural outcome of a null model of mate encounters. Entomologia Experimentalis Et Applicata. 2013; 146(1):26–37. doi: 10.1111/j.1570-7458.2012.01296.x

[77] Mrosovsky N, Provancha J. Sex-ratio of hatchling loggerhead sea turtles—data and estimates from a 5-year study. Canadian Journal of Zoology-Revue Canadienne De Zoologie. 1992; 70(3): 530–8. doi: 10.1139/z92-080

[78] LeBlanc AM, Drake KK, Williams KL, Frick MG, Wibbels T, Rostal DC. Nest Temperatures and hatchling sex ratios from loggerhead turtle nests incubated under natural field conditions in Georgia, United States. Chelonian Conservation and Biology. 2012;11(1):108–16

[79] Sonmez B, Turan C, Ozdilek SY, Turan F. Sex determination of green sea turtle (*Chelonia mydas*) hatchlings on the bases of morphological characters. Journal of the Black Sea/Mediterranean Environment. 2016 p. 93–102.

[80] Delgado C, Canario AVM, Dellinger T. Sex ratios of loggerhead sea turtles *Caretta caretta* during the juvenile pelagic stage. Marine Biology. 2010; 157(5): 979–990. doi: 10.1007/s00227-009-1378-8

